# Early comprehensive care of preterm infants—effects on quality of life, childhood development, and healthcare utilization: study protocol for a cohort study linking administrative healthcare data with patient reported primary data

**DOI:** 10.1186/s12887-016-0640-8

**Published:** 2016-07-22

**Authors:** Jochen Schmitt, Katrin Arnold, Diana Druschke, Enno Swart, Xina Grählert, Ulf Maywald, Andreas Fuchs, Andreas Werblow, Maryan Schemken, Jörg Reichert, Mario Rüdiger

**Affiliations:** Center for Evidence-based Healthcare, University Hospital and Medical Faculty Carl Gustav Carus, TU Dresden, Fetscherstr. 74, 01307 Dresden, Germany; Institute of Social Medicine and Health Economics (ISMG), Faculty of Medicine, Otto-von-Guericke-University Magdeburg, Leipziger Str. 44, 39120 Magdeburg, Germany; Coordination Centre for Clinical Trials, Medical Faculty Carl Gustav Carus, TU Dresden, Fetscherstr. 74, 01307 Dresden, Germany; AOK PLUS, Sternplatz 7, 01067 Dresden, Germany; Department for Neonatology and Pediatric Intensive Care, University Hospital Carl Gustav Carus, TU Dresden, Fetscherstr. 74, 01307 Dresden, Germany

**Keywords:** Early comprehensive care, Preterm infants, Quality of life, Cohort study, Data linkage, Claims data

## Abstract

**Background:**

About 9 % of all children in Germany are born preterm. Despite significant improvements of medical care, preterm infants are at a greater risk to develop short and long term health complications. Negative consequences of preterm birth include neurodevelopmental disabilities, behavioral problems or learning disorders. Most data on effects of prematurity are derived from single or multi-center studies and not population-based.

Since some of the long term problems of preterm delivery are associated with a disturbed parent-child interaction originating in the neonatal period, several intervention programs became available aiming to strengthen the early parent-child relationship. However, there is insufficient knowledge regarding the psychosocial and socioeconomic impact of these interventions. Prior to introducing them into routine care, those effects have to be rigorously evaluated.

The population-based cohort study EcoCare-PIn (Early comprehensive Care of Preterm Infants—effects on quality of life, childhood development, and healthcare utilization) will investigate the following primary research questions: 1) What are the short- and long-term consequences of preterm birth with regard to parental stress, parent-child relationship, childhood development, quality of life and healthcare utilization including costs?

2) Does early family-centered psychosocial care prevent the hypothesized negative consequences of preterm birth on the above mentioned outcomes?

**Methods/Design:**

EcoCare-PIn examines the research questions by means of a linkage of a) pseudonymized administrative individual-level claims data from the German statutory health insurance AOK PLUS on approximately 140,000 children born between 2007 and 2013 in Saxony, and b) primary data collected from the parents/caregivers of all very low birth weight (<1,500 g; *n* = 1,000) and low birth weight infants (1,500 to 2,500 g; *n* = 5,500) and a matched sample of infants above 2,500 g birth weight (*n* = 10,000).

**Discussion:**

In Saxony, approximately 50 % of all individuals are insured at the AOK PLUS. The linkage of patient-level administrative and primary data is a novel approach in neonatal research and probably the only way to overcome shortcomings of studies solely relying on one data source. The study results are based on an observation period of up to 8 years and will directly inform perinatal healthcare provision in Saxony and Germany as a whole.

## Background

About 9 % of all infants are born prematurely, i.e. prior to completing 37 weeks of gestation [1, 2]. Advances in perinatal care during the last decades resulted in a significantly decreased mortality of preterm infants and a reduction of severe organ damage [3, 4]. Nevertheless, preterm born children are still at a higher risk of long-term health and developmental problems compared to their term born counterparts. Known consequences of preterm birth include but are not limited to motor or sensory impairments, learning disabilities or behavioral problems [5–12]. These disturbances bear the potential to compromise the quality of life of affected children and families [13, 14], but also constitute a substantial burden for the healthcare system [15–17]. Whereas impairment can be explained to some extent by organ damage, great variations in development are found despite a similar pattern of injury [18].

Socioeconomic parameters and the quality of the parent-child-relationship have been identified as important determinants of the long-term development of children [19–21]. Especially the parent-child-relationship is significantly affected by preterm delivery, since the immaturity of the newborn interferes with the physiological process of postnatal bonding and attachment. Starting before birth, the complex process of attachment is an essential part of the parental behavioral system that prepares adults for caregiving [22]. It can be disturbed by the premature interruption of pregnancy, an early separation of the newborn from the parents due to necessary medical treatments and the immature babies’ limited attachment behaviors during the first months of life (e.g. smiling, babbling). These circumstances make it more difficult for parents to affectively interact with their child [23, 24] and therefore represent a significant barrier to the development of a well-functioning parent-child relationship early in life [25].

High levels of parental stress, which have frequently been reported when children are born preterm [26–28], are another risk factor for a healthy child development [29].

Existing evidence on the consequences of preterm birth is strongly based on single or multicenter studies that investigated a small selection of outcomes and/or are focused on special groups of preterm infants. For a more comprehensive picture, there is a need for large-scale population-based studies that include all infants regardless of gestational age and investigate the consequences of preterm birth over a substantial time period for affected children, families and the healthcare system. Studies that link individual-level questionnaire data and administrative health insurance data offer great opportunities for the investigation of the broad spectrum of clinical, psychosocial and socioeconomic outcomes of preterm birth, but are still novel in the field.

### Family-centered care program (FamilieNetz)

Following the increased knowledge about the adverse consequences of preterm birth on child and family, but also on the healthcare system, different psychosocial interventions for parents of preterm infants have been developed with the aim to improve the early parent-child-relationship and reduce parental stress [30–35]. However, existing interventions in Germany are not standardized and have not been evaluated rigorously, making it difficult to draw firm conclusions on their preventive effects [36–38].

A novel support and training program for families of sick newborns and preterm infants (FamilieNetz) was developed and introduced in clinical routine at the Department of Neonatology and Pediatric Intensive Care at the University Hospital Carl Gustav Carus, Dresden in 2009. The comprehensive program consists of different interventions, including parent counseling, parent education and discharge planning. Its philosophy focuses on empowering parents in primary caregiving for their child and involving them in daily routine as early as possible by giving them psychological and social-medical support during the initial hospital stay [39].

Single components of the intervention program have been evaluated and as a consequence health insurance companies in Saxony are compensating it in part since 2012. In a next step, the program shall be introduced into other perinatal centers in Saxony and later in Germany as well. However, prior to the introduction, a comprehensive evaluation of the whole multiple component approach is required, in accordance with the principles of evidence-based healthcare.

In order to address the issues outlined above, the study EcoCare-PIn will examine the following research questions:

1. What are the short- and long-term consequences of preterm birth with regard to parental stress, parent-child relationship, family and child quality of life, child development, and healthcare utilization including costs?
2. Does early family-centered psychosocial care prevent the hypothesized negative consequences of preterm birth on parental stress, parent-child relationship, family and child quality of life, child development, and healthcare utilization including costs?

#### Hypotheses

1. Preterm birth and subsequent need for medical treatment disturbs the process of postnatal attachment and will have an impact on (I) parental stress, parent-child-relationship, and quality of life of family and child at age 1–7 years (primary outcomes) and (II) mental and physical child development (e.g. school performance, comorbidities), healthcare utilization and cost (secondary outcomes) during that time of life.
2. Early psychosocial intervention that aims to support attachment and parental competency improves the parent-child-relationship and modifies the hypothesized adverse short and long-term consequences of preterm birth for the child, the child’s family, and the healthcare system.

**Fig. 1:**
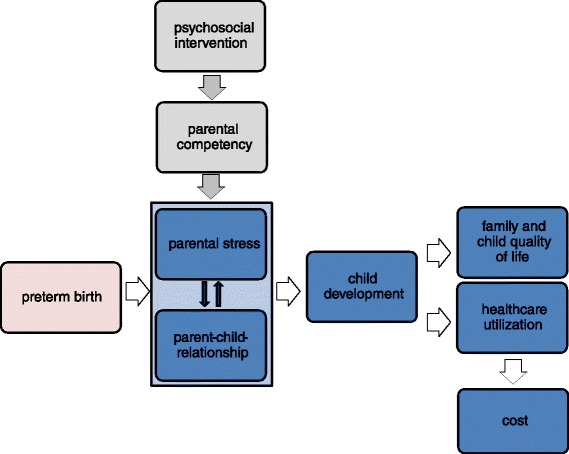
Hypothesized causal model (adapted from: Reichert & Rüdiger, 2012 [39])

The hypothesized causal model is shown in Fig. 1.

## Methods/Design

EcoCare-PIn is a cohort study comprising the linkage of individual-level data from two different sources: (1) administrative claims data from the German statutory health insurance AOK PLUS on all insured children born between 2007 and 2013 in Saxony and (2) primary data collected by means of questionnaires (in 2014/2015) from parents or legal caregivers of VLBW (very low birth weight) infants (<1,500 g), LBW (low birth weight) infants (1,500 to 2,499 g), and infants with a birth weight of 2,500 g or higher as controls.

Preterm birth is generally defined as birth before 37 completed weeks of gestation [2]. As there is considerable overlap between gestational age under 37 weeks and birth weight below 2,500 g, the use of birth weight (<2,500 g) for the definition of preterm born children instead of gestational age (<37 weeks) can be regarded as an acceptable approach in case that data on gestational age are not available or of uncertain quality [40, 41]. As the AOK PLUS does not provide data on gestational age, but on birth weight, we will use the latter as proxy measure for preterm birth.

### Design and recruitment

The study is based on pseudonymized *administrative outpatient and inpatient data* of all children born in Saxony between January 1^st^, 2007 and December 31^st^, 2013 with health insurance at the AOK PLUS. The whole cohort of newborns will include almost 140,000 children, with approximately 1,000 VLBW- and 5,500 LBW-children among them. A broad set of insurance data will be provided and analyzed within this study, including birth weight (classified into <1,500 g (VLBW), 1,500–2,499 g (LBW) and ≥2,500 g (controls)), selected sociodemographic characteristics of children (age, sex, first three digits of ZIP code), diagnoses (ICD-10 codes), diagnosis related billing information (DRG-codes), prescriptions (PZN-codes, ATC-codes, cost), medical procedures (OPS-codes), claim codes for outpatient services and procedures (EBM-codes) and data of healthcare providers (discipline, first three digits of ZIP code) [42]. Based upon these pseudonymized administrative data, we will investigate and compare VLBW-, LBW- and control children with regard to mental and physical development, healthcare utilization and cost from birth through December 31^st^, 2013.

To enable further analyses, additional *primary data* will be collected on a subgroup of the described cohort of children. With support from the AOK PLUS, parents or legal caregivers from all preterm born children and parents/legal caregivers of a matched sample of 10,000 control children will be approached via postal questionnaires. Control children for the primary data collection will be selected via frequency matching according to birth year, sex and administrative district.

The questionnaire will cover information on parental stress, parent-child relationship, child’s physical and mental development (including school performance), quality of life of children, family quality of life, sociodemographic characteristics of parents, and exposure to psychosocial care in the neonatal period. These primary data will be linked to the administrative data on an individual level.

**Fig. 2:**
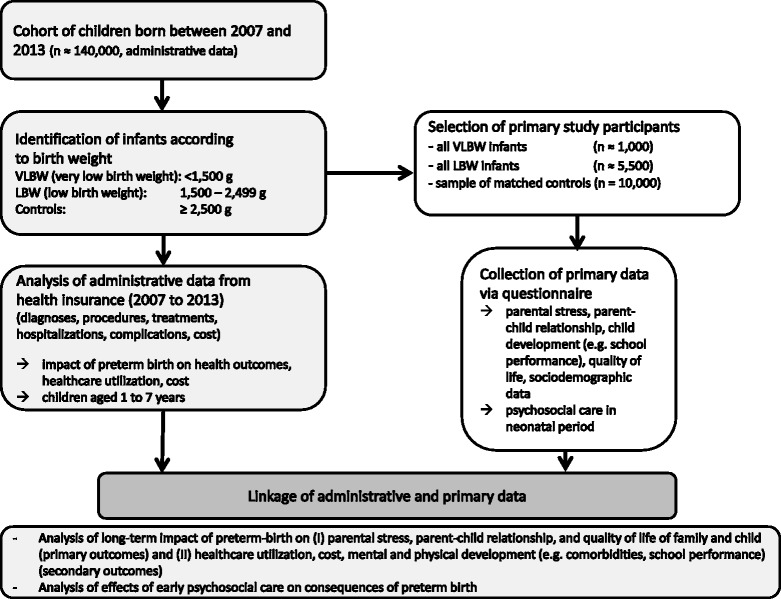
Study flow chart

Figure 2 provides a flow chart on the design of the study.

### Instruments

The following instruments will be used as part of the primary data collection to assess parental stress, parent-child-relationship, mental development and quality of life. These tests are widely used, valid, reliable, and available in German language:

- parental stress and parentchild relationship
  - EBI [Eltern-Belastungs-Inventar] (german version of PSI [Parenting stress index]); Tröster H 2011 [43].
- mental development of children
  - SDQ [Strengths and Difficulties Questionnaire]; Goodman R 1997 [44].
- quality of life
  - parents: WHOQOL-BREF [WHO-Quality of Life—brief version]; Angermeyer MC et al. 2000 [45].
  - children: KINDL [Questionnaire for the collection of health-related quality of life in children and adolescents]; Ravens-Sieberer U, Bullinger M 1998 [46].

These instruments are incorporated into the study questionnaire after having obtained the respective author licenses. At the timepoint of submission of this study protocol, all necessary licenses had been obtained.

To meet the goal of assessing potential consequences of preterm birth not covered with these instruments (e.g. school performance, details on physical development) as well as details on psychosocial care in the neonatal period, the study questionnaire will also contain items developed by the authors and the scientific advisory board. The study questionnaire will be beta-tested and revised if necessary prior to application.

### Procedures of data collection

The fundamental principle of the data concept, which will be outlined below, is to strictly separate the sites of data collection from the data analysis site.

Starting point is the site of data collection I (AOK PLUS), which submits pseudonymized (pseudonym I) outpatient and inpatient data of all children born in Saxony between January 2007 and December 2013 with health insurance at the AOK PLUS to the data analysis site (Center for Evidence-Based Healthcare (ZEGV)).

Data analysis site (ZEGV) will then identify individuals eligible for the primary data collection and gives pseudonyms (pseudonym I) of all VLBW and LBW children as well as of matched control children to the site of data collection I (AOK PLUS). For reasons of better practicability, ZEGV additionally transfers a new short pseudonym per individual (pseudonym II).

Data collection site I (AOK PLUS) de-pseudonymizes these information and sends questionnaires with pseudonym I and II to the parents or legal caregivers of the selected AOK PLUS members. Each questionnaire is supplemented by a) a detailed study information explaining study objectives, study design and data protections issues, and b) an informed consent form.

AOK PLUS members whose caregivers agree with study participation send the completed questionnaire and the signed consent form to the data collection site II (KKS).

The data collection site II (KKS) will receive the completed questionnaires and consent forms. Only questionnaires accompanied by a validly signed consent form will be considered for the study. KKS will delete any personal identifiers from those datasets, pseudonymize them and send them to the data analysis site (ZEGV) for the linkage with administrative data and statistical analysis. Throughout the study, the site of data analysis (ZEGV) will only use pseudonymized data.

**Fig. 3:**
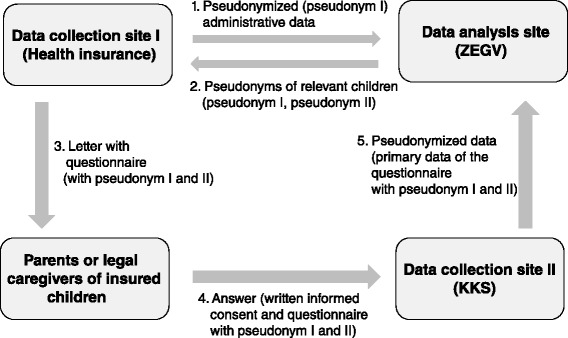
Data collection and data flow

The process of data collection is summarized in Fig. 3.

### Data preparation

The classification into different birth weight groups (VLBW, LBW and controls) is based on documented birth weight and assigned directly by the health insurance company. In the absence of a documented birth weight, ‘unknown birth weight’ is encoded by the insurance. In order to limit the number of children in this group, those children will be reassigned by the authors according to the child`s inpatient or outpatient P07-ICD-diagnoses which directly refer to a specific birth weight (ICD-10-GM P07.0-, P07.00, P07.01, P07.02, P07.10 or P07.11^1^^a ^lead to reassignment to the VLBW-group; P07.1- or P07.12^b^ lead to reassignment to the LBW-group).

In case there is none of the mentioned birth weight specific diagnoses it will be checked whether a hospital admission within the first 7 days after birth has been coded and a documented admission weight is available. If both criteria are fulfilled, the child will be reassigned in accordance with the given admission weight to the VLBW, LBW or control group.

### Statistical analysis

**Fig. 4:**
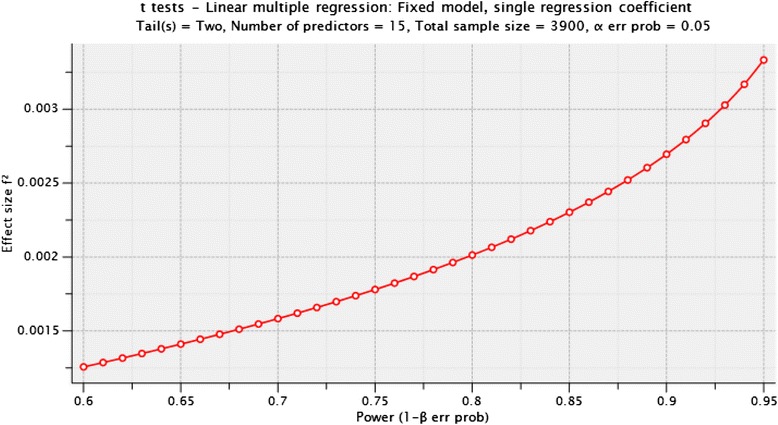
Detectable effect sizes (Cohen’s f^2^) in analyses with the linked dataset depending on expected power

Multivariate binary or multinomial logistic regression models as well as multiple linear regression models will be used to analyze the effect of birth weight (differentiated into VLBW, LBW and birth weight ≥ 2,500 g) on the outcomes. Adjustment will be made for sex, year of birth and socioeconomic status. Modification of the presumed adverse short and long-term effects of preterm birth through early psychosocial care will be analyzed by modeling the exposure to such a program as predictor for parental stress, parent-child relationship, quality of life, child development, healthcare utilization and costs. Effect modification will be explored by including the corresponding interaction-terms in the regression models. With an expected response rate of 30 % and an expected power (1-β) of 80 %, effect sizes (Cohen’s f) of 0.045 or higher can be detected at a 5 % significance level in analyses with the linked dataset (see Fig. 4). For analyses based on the whole administrative data cohort, effect sizes (Cohen’s f) of 0.035 or higher can be detected with a power of 80 % at the 5 % significance level.

In an administrative data based cohort study the quality of data depends on the quality of documentation in care as well as on the quality of data recording by AOK PLUS [47]. Missing data in questionnaires (primary data collection) will be handled by methods of imputation or partial imputation if recommended in the test manuals. Statistical analyses will be conducted using the programmes STATA and SPSS.

### Ethical and legal considerations

In this project, linkage of data from different sources is conducted solely for AOK PLUS insured individuals whose parents or legal caregivers have given their written informed consent. A positive internal evaluation of the Privacy Commissioner of the AOK PLUS was performed. The methods and procedures of the study are developed in compliance with the ethical principles of the Declaration of Helsinki [48] and the guidance provided in Good Epidemiologic Practice [49] in connection with Good Practice Secondary Data Analysis [47]. The study has been approved by the responsible ethics committee of the Technische Universität Dresden (reference number EK 67022014) and the Saxon Data Protection Commissioner (reference number 2-7410-74/1).

The authors are experienced in developing and realizing data protection concepts linking primary and administrative data [50]. In addition, Standard Operating Procedures (SOPs) will be developed to guarantee the fulfilment of best practice requirements in the studies’ data linkage. Quality assurance of the study is carried out by the Institute of Social Medicine and Health Economics of the Otto-von-Guericke-University Magdeburg.

## Discussion

Our study comprehensively investigates the short- and long-term consequences of premature birth and examines the effects of specialized family-centered care approaches to minimize the adverse long-term consequences with respect to child development, quality of life and healthcare utilization including costs. This rigorous, population-based evaluation is necessary in consideration of the currently existing evidence gaps mentioned above.

The AOK PLUS has confirmed the support of the project in a letter of intent. The use of administrative data of all children insured at the AOK PLUS and the fact that this health insurance covers about 50% of individuals in the study region allows for a high generalizability of the study results. Linking the individual-level administrative data of an entire birth-cohort with primary data on an individual level represents a new and promising approach for neonatal research. As the planned linkage includes a broad set of “objective” administrative and “subjective” primary data, the study will provide an excellent evidence base for the further development and implementation of value-based perinatal health care in regional settings.^2^

Findings from this study will help to further improve care programs and provide valuable evidence for decision makers to implement effective comprehensive preventive programs into the German healthcare system. As indicated by the Saxony ministry of social affairs this study will directly inform health policy and decision making in neonatal care.

## Abbreviations

ATC-Code, anatomical therapeutic chemical-classification; DRG-Code, Diagnosis Related Groups; EBM-Code, claim codes for outpatient services and procedures; ICD-10-GM, International Statistical Classification of Diseases and Related Health Problems, 10th revision, German Modification; LBW, low birth weight (1,500–2,499 g); OPS-Code, Code according to Operations and Procedures Coding System (OPS); PZN-Code, pharmaceutical central number; VLBW, very low birth weight (<1,500 g).

## Footnotes

1 ^a^ ICD-10-GM P07.0- newborn: birth weight 999 g or less; P07.00 newborn: birth weight below 500 g; P07.01 newborn: birth weight 500–749 g; P07.02 newborn: birth weight 750–999 g; P07.10 newborn: birth weight 1,000-1,249 g; P07.11 newborn: birth weight 1,250-1,499 g. ^b^ ICD-10-GM P07.1- newborn: birth weight 1,000-2,499 g; P07.12 newborn: birth weight 1,500-2,499 g

2 Value-based healthcare, as suggested by Porter [51, 52], aims at providing specialized, cross-sectoral healthcare while optimizing the ratio of patient-relevant benefits and costs across the entire chain of medical care. Main precondition is the assessment of both patient-relevant data on the effects of an intervention (e.g. on quality of life) as well as “objective” economic data within a regional setting.

## References

[1] AQUA (2015). Institut für angewandte Qualitätsförderung und Forschung im Gesundheitswesen GmbH. Bundesauswertung zum Verfahrensjahr 2014 16/1—Geburtshilfe.

[2] Howson CP, Kinney MV, Lawn JE, editors. March of Dimes, PMNCH, Save the Children, World Health Organization. Geneva: Born too soon: The global action report on preterm birth. World Health Organization; 2012.

[3] Landmann E, Misselwitz B, Steiss JO, Gortner L (2008). Mortality and morbidity of neonates born at < 26 weeks of gestation (1998–2003). A population-based study. J Perinat Med.

[4] Patel RM, Kandefer S, Walsh MC, Bell EF, Carlo WA, Laptook AR (2015). Causes and timing of death in extremely premature infants from 2000 through 2011. N Engl J Med.

[5] Aarnoudse-Moens CSH, Weisglas-Kuperus N, van Goudoever JB, Oosterlaan J (2009). Meta-analysis of neurobehavioral outcomes in very preterm and/or very low birth weight children. Pediatrics.

[6] Adams JN, Feldman HM, Huffman LC, Loe IM (2015). Sensory processing in preterm preschoolers and its association with executive function. Early Hum Dev.

[7] Institute of Medicine (US) Committee on Understanding Premature Birth and Assuring Healthy Outcomes: Preterm Birth: Causes, Consequences, and Prevention. Edited by Behrman RE, Butler AS. Washington: National Academies Press; 2007. 20669423

[8] Bhutta AT, Cleves MA, Casey PH, Cradock MM, Anand KJS (2002). Cognitive and behavioral outcomes of school-aged children who were born preterm - a meta-analysis. JAMA.

[9] Damm G, Macha T, Petermann F, Voss W, Sens B (2015). Qualitätsanalysen zur Entwicklung Frühgeborener: Ergebnisse des niedersächsischen Frühgeborenen-Nachuntersuchungsprojekts und eines Vergleichskollektivs reif geborener Kinder. Z Evid Fortbild Qual Gesundhwes.

[10] Johnson S, Fawke J, Hennessy E, Rowell V, Thomas S, Wolke D (2009). Neurodevelopmental disability through 11 years of age in children born before 26 weeks of gestation. Pediatrics.

[11] Kerr-Wilson CO, MacKay DF, Smith GCS, Pell JP (2012). Meta-analysis of the association between preterm delivery and intelligence. J Public Health.

[12] Lindstrom K, Lindblad F, Hjern A (2011). Preterm birth and attention-deficit/hyperactivity disorder in schoolchildren. Pediatrics.

[13] Zwicker JG, Harris SR, Klassen AF (2013). Quality of life domains affected in children with developmental coordination disorder: a systematic review. Child Care Health Dev.

[14] Vieira MEB, Linhares MBM (2011). Developmental outcomes and quality of life in children born preterm at preschool- and school-age. J Pediatr (Rio J).

[15] Mac Bird T, Bronstein JM, Hall RW, Lowery CL, Nugent R, Mays GP (2010). Late preterm infants: birth outcomes and health care utilization in the first year. Pediatrics.

[16] Petrou S (2005). The economic consequences of preterm birth during the first 10 years of life. Br J Obstet Gynaecol.

[17] Reichert J, Schemken M, Manthei R, Steinbronn R, Bucher U, Albrecht M (2013). Health insurance expenses for children in the first 5 years of life—a cohort-based analysis. Z Evid Fortbild Qual Gesundhwes.

[18] Wolke D (2006). Psychological development of extremely preterm (<26 weeks) and not so preterm (27–35 weeks) children—the big picture. Early Hum Dev.

[19] Forcada-Guex M, Pierrehumbert B, Borghini A, Moessinger A, Muller-Nix C (2006). Early dyadic patterns of mother-infant interactions and outcomes of prematurity at 18 months. Pediatrics.

[20] Poehlmann J, Schwichtenberg AJM, Bolt DM, Hane A, Burnson C, Winters J (2011). Infant physiological regulation and maternal risks as predictors of dyadic interaction trajectories in families with a preterm infant. Dev Psychol.

[21] Schmitt J, Rossa K, Rüdiger M, Reichert J, Schirutschke M, Frank H (2015). Determinanten der psychischen Gesundheit im Einschulungsalter. Ergebnisse einer populationsbezogenen Untersuchung in Dresden. Kinder- und Jugendarzt.

[22] Landsem IP, Handegard BH, Tunby J, Ulvund SE, Ronning JA. Early intervention program reduces stress in parents of preterms during childhood, a randomized controlled trial. Trials. 2014;15:387. 10.1186/1745-6215-15-387 PMC4198672 25282345

[23] Brisch KH, Bechinger D, Betzler S, Heinemann H, Kachele H, Pohlandt F (2005). Attachment quality in very low-birthweight premature infants in relation to maternal attachment representations and neurological development. Parenting-Sci Pract.

[24] Grossmann KE, Grossmann K (2015). editors. Bindung und menschliche Entwicklung. John Bowlby, Mary Ainsworth und die Grundlagen der Bindungstheorie.

[25] Korja R, Latva R, Lehtonen L (2012). The effects of preterm birth on mother-infant interaction and attachment during the infant’s first 2 years. Acta Obstet Gynecol Scand.

[26] Schappin R, Wijnroks L, Uniken Venema MMAT, Jongmans MJ. Rethinking stress in parents of preterm infants: a meta-analysis. PLoS ONE 2013;8:E54992. 10.1371/journal.pone.0054992 PMC3566126 23405105

[27] Singer LT, Fulton S, Kirchner HL, Eisengart S, Lewis B, Short E (2010). Longitudinal predictors of maternal stress and coping after very low-birth-weight birth. Arch Pediatr Adolesc Med.

[28] Treyvaud K, Lee KJ, Doyle LW, Anderson PJ (2014). Very preterm birth influences parental mental health and family outcomes seven years after birth. J Pediatr.

[29] Ong LC, Chandran V, Boo NY (2001). Comparison of parenting stress between Malaysian mothers of four-year-old very low birthweight and normal birthweight children. Acta Paediatr.

[30] Huning BM, Reimann M, Beerenberg U, Stein A, Schmidt A, Felderhoff-Muser U (2012). Establishment of a family-centred care programme with follow-up home visits: implications for clinical care and economic characteristics. Klin Padiatr.

[31] Huppertz C, Gharavi B, Schott C, Linderkamp O (2005). Individuelle, entwicklungsfördernde Pflege basierend auf dem Newborn Individualized Developmental Care and Assessment Program (NIDCAP). Kinderkrankenschwester.

[32] Jiang SY, Warre R, Qiu XM, O’Brien K, Lee SK (2014). Parents as practitioners in preterm care. Early Hum Dev.

[33] Panagl A, Kohlhauser C, Pollak A (2005). Integrative psychologische Elternbetreuung auf der neonatologischen Intensivstation: Konzept und erste Erfahrungen. Z Geburtshilfe Neonatol.

[34] Vonderlin E, Noecker-Ribaupierre M, Wilken M (2010). Supportive interventions in newborn. German newborn intensive care units. Monatsschr Kinderheilkd.

[35] Brisch KH, Bechinger D, Betzler S, Heinemann H (2003). Early preventive attachment-oriented psychotherapeutic intervention program with parents of a very low birthweight premature infant: results of attachment and neurological development. Attach Hum Dev.

[36] Benzies KM, Magill-Evans JE, Hayden KA, Ballantyne M. Key components of early intervention programs for preterm infants and their parents: a systematic review and meta-analysis. BMC Pregnancy and Childbirth 2013;13 Suppl 1:S10. 10.1186/1471-2393-13-S1-S10 PMC3561170 23445560

[37] Brecht CJ, Shaw RJ, St John NH, Horwitz SM (2012). Effectiveness of therapeutic and behavioral interventions for parents of low-birth-weight premature infants: a review. Infant Mental Health J.

[38] Evans T, Whittingham K, Sanders M, Colditz P, Boyd RN (2014). Are parenting interventions effective in improving the relationship between mothers and their preterm infants?. Infant Behav Dev.

[39] Reichert J, Rüdiger M (2012). Psychologisch-sozialmedizinische Versorgung von Eltern Frühgeborener.

[40] Lack N, Zeitlin J, Krebs L, Kunzel W, Alexander S (2003). Methodological difficulties in the comparison of indicators of perinatal health across Europe. Eur J Obstet Gynecol Reprod Biol.

[41] Schneider D, Radloff S, Muller S, Moller D, Bolz M, Briese V. Analysis of infants based on data from the German Perinatal Survey of the years 1994–2011 in Mecklenburg-Pomerania, Germany. Classification of infants with regard to their gestation duration and birth weight. Z Geburtshilfe Neonatol.2015;219:93–98. 10.1055/s-0034-1383584 25525815

[42] Swart E, Ihle P, Gothe H, Matusiewicz D (2014). Routinedaten im Gesundheitswesen: Handbuch Sekundärdatenanalyse: Grundlagen, Methoden und Perspektiven.

[43] Tröster H (2011). Eltern-Belastungs-Inventar (EBI). Deutsche Version des Parenting Stress Index (PSI) von R.R. Abidin.

[44] Goodman R (1997). The strengths and difficulties questionnaire: a research note. J Child Psychol Psychiatry.

[45] Angermeyer MC, Kilian R, Matschinger H (2000). WHOQOL-100 und WHOQOL-BREF. Handbuch für die deutschsprachige Version der WHO Instrumente zur Erfassung von Lebensqualität.

[46] Ravens-Sieberer U, Bullinger M (1998). Assessing health-related quality of life in chronically ill children with the German KINDL: first psychometric and content analytical results. Qual Life Res.

[47] Swart E, Gothe H, Geyer S, Jaunzeme J, Maier B, Grobe TG (2015). Good practice of secondary data analysis (GPS): guidelines and recommendations third revision 2012/2014. Gesundheitswesen.

[48] World Medical Association. World Medical Association Declaration of Helsinki: Ethical Principles for Medical Research Involving Human Subjects. JAMA 2013;310:2191–4. 10.1001/jama.2013.281053 24141714

[49] Deutsche Arbeitsgemeinschaft für Epidemiologie (DAE). Leitlinien und Empfehlungen zur Sicherung von Guter Epidemiologischer Praxis (GEP). 2004. http://www.gesundheitsforschung-bmbf.de/_media/Empfehlungen_GEP.pdf. Accessed 15 July 2016.

[50] Swart E, Thomas D, March S, Salomon T, von der Knesebeck O (2011). Experience with the linkage of primary and secondary claims data in an intervention trial. Gesundheitswesen.

[51] Porter ME (2009). Health care 2009: a strategy for health care reform—toward a value-based system. N Engl J Med.

[52] Porter ME (2010). What is value in health care?. N Engl J Med.

